# The evolutionary arms race on the Z-form-nucleic- acid-binding protein 1 front: endogenous Z-RNA as a self-danger signal

**DOI:** 10.1186/s43556-026-00404-9

**Published:** 2026-01-27

**Authors:** Yuxuan Tang, Shenghui Niu, Lin Zhao

**Affiliations:** Key Laboratory of Birth Defects and Related Diseases of Women and Children, Department of Paediatrics, West China Second University Hospital, State Key Laboratory of Biotherapy and Collaborative Innovation Center of Biotherapy, Sichuan University, Chengdu, 610041 China

In a recent study published by Yin et al., viral infection was shown to activate ZBP1 predominantly through host-derived Z-RNAs generated by virus-induced disruption of transcription termination, rather than through viral Z-RNAs themselves [1]. This finding redefines ZBP1 as a sensor of infection-induced cellular dysfunction, revealing implications for understanding antiviral surveillance and inflammatory outcomes during infection.

The conventional model proposes that Z-form-nucleic-acid-binding protein 1 (ZBP1) recognizes viral RNAs generated during IAV replication, which may adopt a Z-conformation due to aberrant replication dynamics or specific double-stranded RNA structures [2]. However, the precise ligands responsible for ZBP1 activation had not been systematically defined. Using extensive sequencing and integrative analyses, Yin et al. comprehensively mapped the origins of Z-RNAs during viral infection and demonstrated that viruses such as HSV-1 and IAV disrupt host transcription termination through ICP27 and NS1, respectively [1]. This interference induces readthrough transcription of endogenous retroelements, generating aberrantly elongated host transcripts that fold into Z-RNA structures. Crucially, these virus-induced, host-derived “self” Z-RNAs—rather than viral “non-self” RNAs—constitute the dominant ligands activating ZBP1 (Fig. 1).

**Fig. 1:**
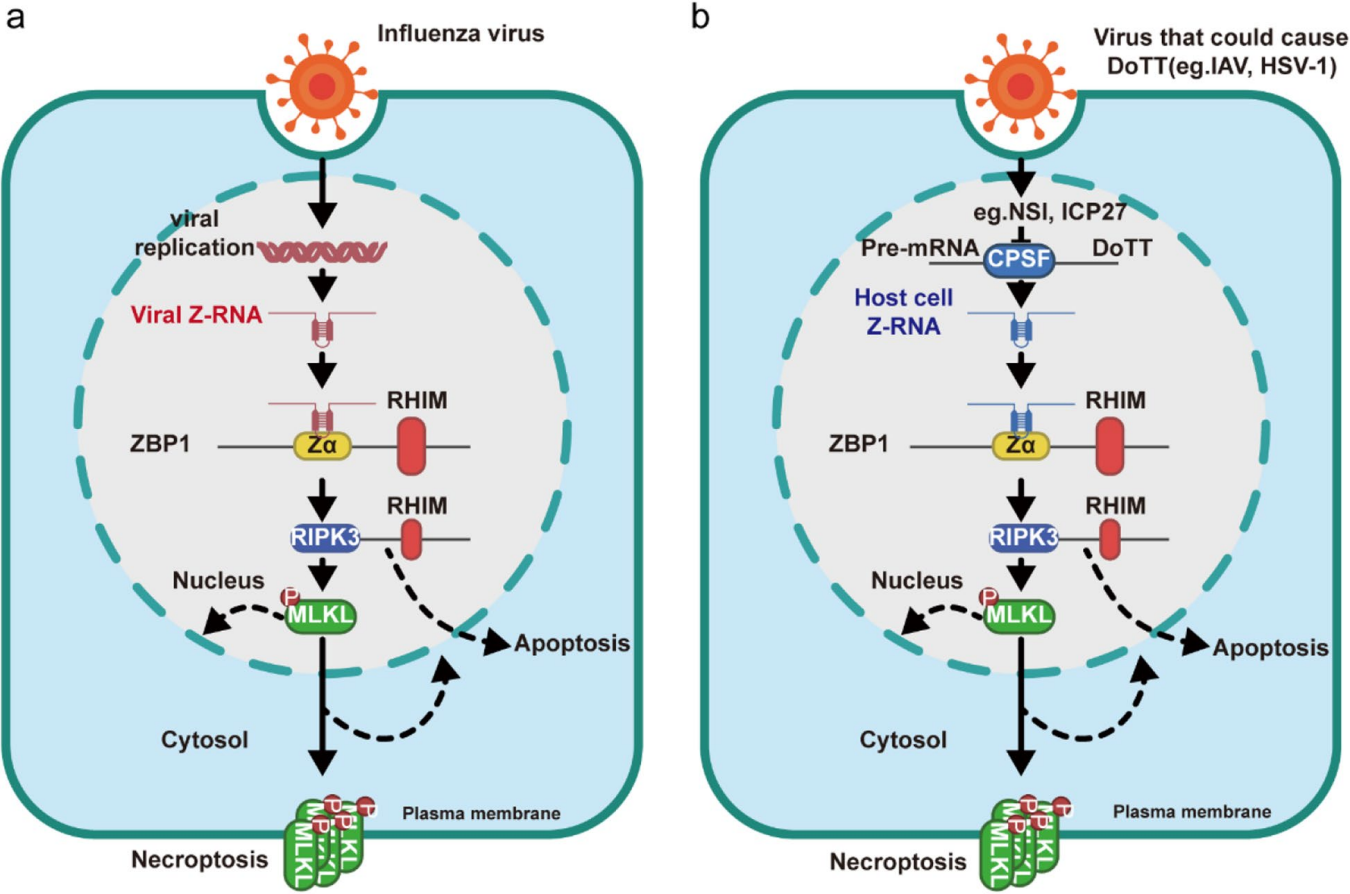
Reevaluating the ZBP1 activation pathway upon viral infection. **a** Canonical model of ZBP1 activation by viral Z-RNA. In the traditional paradigm, ZBP1 is activated through the direct recognition of Z-form viral nucleic acids generated during viral replication. Upon influenza virus (IAV) infection, aberrant viral RNA replication or the formation of double-stranded RNA intermediates was proposed to give rise to viral Z-RNA species. These Z-form viral RNAs are sensed by the Zα domain of ZBP1, leading to ZBP1 activation. Activated ZBP1 subsequently engages RIPK3 through homotypic RHIM–RHIM interactions, resulting in RIPK3 phosphorylation and downstream phosphorylation of MLKL. Phosphorylated MLKL oligomerizes and translocates to the plasma membrane, ultimately executing necroptotic cell death. In parallel, ZBP1–RIPK3 signaling can also intersect with apoptotic pathways, as indicated by dashed arrows. In this model, viral nucleic acids themselves constitute the primary danger signal that initiates ZBP1-dependent inflammatory cell death. **b** Revised model: ZBP1 senses virus-induced cellular dysfunction via endogenous Z-RNA. The updated model proposes that ZBP1 activation is driven predominantly by host-derived Z-RNAs generated as a consequence of viral perturbation of cellular transcriptional homeostasis. Viruses such as IAV and express proteins (e.g., NS1) that interfere with host transcription termination machinery, including CPSF, thereby inducing disruption of transcription termination (DoTT). This transcriptional dysregulation results in extensive readthrough transcription of endogenous retroelements which contains reverse repetitive genomic regions, producing aberrantly elongated host transcripts that adopt the Z-RNA conformation. These endogenous Z-RNAs are subsequently recognized by the Zα domain of ZBP1. Similar to the canonical pathway, ZBP1 activation promotes RHIM-dependent recruitment of RIPK3, leading to RIPK3 and MLKL phosphorylation, MLKL oligomerization, and necroptotic plasma membrane permeabilization, with potential crosstalk to apoptotic signaling pathways. In this revised framework, ZBP1 functions primarily as a sensor of infection-induced cellular abnormalities rather than as a direct detector of viral nucleic acids

The host has established a more formidable “bottom-line” alarm system by detecting signals of its own abnormalities. Unlike traditional “non-self” recognition, this mechanism monitors the cellular state disruption inevitably triggered by viral activity, making it mechanistically difficult for viruses to evade detection. Viruses cannot prevent alarm triggering unless they abandon highly efficient replication strategies (such as transcriptional hijacking). This imposes powerful new selective pressures, compelling viruses to evolve more potent downstream signaling suppression capabilities rather than relying solely on simple ligand mimicry.

Confronted by ZBP1 as a potent sentinel, viruses have evolved multi-layered evasion strategies covering the entire signaling cascade, to respond to danger signals, including endogenous Z-RNA induced by the organism itself, constituting a remarkable evolutionary arms race [3]. These strategies can be categorized into three tiers: For example, The vaccinia virus E3 protein acts as a molecular decoy in this process. Its Zα domain competitively binds to Z-nucleic acids, thereby sequestering them and competing with ZBP1 for Z-nucleic acid binding. IAV's NS1/NP protein may also utilize its dsRNA-binding domain to shield viral replication intermediates, limiting the exposure of its own Z-RNA. Second, signal transduction interference. Murine cytomegalovirus (MCMV), for example, encodes a protein containing a RHIM domain that effectively disrupts the recruitment of downstream adaptor proteins RIPK3 and RIPK1 by ZBP1 via its own RHIM domains. Third, downstream pathway blockade. Numerous virus express inhibitors targeting the execution phase of cell death. SARS-CoV-2, for instance, encodes the NSP13 protein, which can competitively bind RIPK3 against ZBP1, thereby inhibiting the occurrence of necroptosis, confirming the pathway's central role and efficacy in antiviral immunity.

Particularly sophisticated are the "offense-defense integration" strategies employed by some viruses. Using HSV-1 as an example, its ICP27 protein induces host Z-RNA production ("offense"), while its ICP6 protein acts as a "molecular circuit-breaker" to block death signaling downstream of ZBP1 ("defense") [4]. This precise bidirectional regulation of the same pathway perfectly illustrates the fine-tuned temporal control evolved by viruses to manage the immune consequences of their own disruptive actions, and strongly attests, from an evolutionary perspective, to the immense selective pressure imposed by the Z-RNA-ZBP1 axis.

The differential utilization of this pathway by various viruses fundamentally reflects an evolutionary trade-off between "intervening in core host functions for efficient replication" and "achieving immune stealth for sustained infection." Viruses like vaccinia, whose genomes have an intrinsic potential to generate Z-NA, including both Z-RNA and Z-DNA, during their life cycle, primarily adopt a "shielding" strategy. In contrast, HSV-1 and IAV take an alternative path by hijacking the host transcriptional machinery, turning the accumulation of host-derived Z-RNA into a delayed death trigger. This strategy allows the virus to exploit the multifunctional roles of proteins like NS1 to suppress various nucleic acid sensing pathways, including ZBP1, during early infection, buying a crucial time window for viral replication. As infection progresses and host-derived Z-RNA accumulates beyond a certain threshold, it overcomes the suppression and triggers ZBP1-dependent cell death. This host cell death impedes further viral replication and amplification, thereby limiting viral dissemination and ultimately achieving viral clearance.

Furthermore, for other viruses reported to activate ZBP1 through unclear mechanisms (e.g., SARS-CoV-2), the activation process likely follows a similar evolutionary logic. It may stem from the combined effects of Z-RNA produced during viral replication and cellular stress induced by viral proteins (e.g., ORF6) disrupting host nuclear membrane function [5]. These observations collectively indicate that viral engagement with and evasion of the ZBP1 pathway are not merely determined by simple genomic traits but are the inevitable outcome of an evolutionary trade-off between "replication efficiency" and "immune stealth." Importantly, this trade-off does not imply that ZBP1 activation is an inevitable consequence of viral replication, but rather that viruses adopting more aggressive strategies to reprogram host nucleic acid metabolism face increased selective pressure to counteract ZBP1 signaling. The extent to which this balance is conserved across different virus families, and whether distinct replication strategies converge on host Z-RNA production, remain open questions. Investigating the molecular basis of these differences will provide key insights into the deep logic of host-virus co-evolution.

Overall, the pioneering work of Yin and colleagues demonstrates that Z-RNA, induced by viruses within host cells, acts as a vital endogenous danger signal. This redefines the ZBP1 pathway, shifting its recognized role from detecting external threats ("non-self") to monitoring internal abnormalities ("self-abnormality"). And this finding provides a new conceptual framework for therapeutic intervention that targets the upstream generation, processing, or cellular localization of host Z-nucleic acids, thereby modulating ZBP1 pathway activation independently of downstream cell death machinery. An important future direction is to define how virus-induced host Z-NA production is coordinated with other nucleic acid sensing pathways, such as RIG-I–MAVS and cGAS–STING, which may respond to distinct but temporally overlapping nucleic acid species generated by the same transcriptional and nuclear stress. Dissecting how these pathways share or compete for nucleic acid substrates and signaling thresholds may clarify how cells integrate multiple endogenous danger signals during viral infection. Also, whether ZBP1 serves as a general sensor of endogenous Z-form nucleic acids beyond the two viruses studied here remains to be determined.

## Data Availability

Not applicable.
